# *Escherichia coli *genome-wide promoter analysis: Identification of additional AtoC binding target elements

**DOI:** 10.1186/1471-2164-12-238

**Published:** 2011-05-13

**Authors:** Eleftherios Pilalis, Aristotelis A Chatziioannou, Asterios I Grigoroudis, Christos A Panagiotidis, Fragiskos N Kolisis, Dimitrios A Kyriakidis

**Affiliations:** 1 Institute of Biological Research and Biotechnology, National Hellenic Research Foundation, 48 Vas. Constantinou Ave, GR-11635, Athens, Greece; 2 Laboratory of Biochemistry, Department of Chemistry, Aristotle University of Thessaloniki, GR-54124, Greece; 3 Department of Pharmaceutical Sciences, Aristotle University of Thessaloniki, Thessaloniki, GR-54124, Greece

## Abstract

**Background:**

Studies on bacterial signal transduction systems have revealed complex networks of functional interactions, where the response regulators play a pivotal role. The AtoSC system of *E. coli *activates the expression of *atoDAEB *operon genes, and the subsequent catabolism of short-chain fatty acids, upon acetoacetate induction. Transcriptome and phenotypic analyses suggested that *atoSC *is also involved in several other cellular activities, although we have recently reported a palindromic repeat within the *atoDAEB *promoter as the single, *cis*-regulatory binding site of the AtoC response regulator. In this work, we used a computational approach to explore the presence of yet unidentified AtoC binding sites within other parts of the *E. coli *genome.

**Results:**

Through the implementation of a computational *de novo *motif detection workflow, a set of candidate motifs was generated, representing putative AtoC binding targets within the *E. coli *genome. In order to assess the biological relevance of the motifs and to select for experimental validation of those sequences related robustly with distinct cellular functions, we implemented a novel approach that applies Gene Ontology Term Analysis to the motif hits and selected those that were qualified through this procedure. The computational results were validated using Chromatin Immunoprecipitation assays to assess the *in vivo *binding of AtoC to the predicted sites. This process verified twenty-two additional AtoC binding sites, located not only within intergenic regions, but also within gene-encoding sequences.

**Conclusions:**

This study, by tracing a number of putative AtoC binding sites, has indicated an AtoC-related cross-regulatory function. This highlights the significance of computational genome-wide approaches in elucidating complex patterns of bacterial cell regulation.

## Background

AtoSC is a two-component regulatory system that activates the transcription of the *atoDAEB *operon genes in *E. coli*, the products of which are involved in the catabolism of short-chain fatty acids [1-3]. Acetoacetate has been identified as the inducer of this system. Additionally, modulation of the poly-hydroxybutyrate (cPHB) biosynthesis in *E. coli *by AtoC [1], and the effects of spermidine [2] or histamine [3] on this process, suggest a more critical metabolic role for this system.

The AtoC response regulator shares significant homology and characteristic properties with the NtrC-NifA transcriptional factors that activate sigma-54 RNA polymerase [4]. The AtoC binding site was experimentally verified as an inverted 40-bp palindrome (two identical inverted 20-bp sites), located upstream of the transcription initiation site of the *atoDAEB *operon. These *cis*-elements were found to control both the promoter inducibility by acetoacetate and AtoC binding. Moreover, chromatin immunoprecipitation (ChIP) experiments confirmed *in vivo *acetoacetate-inducible AtoC binding [5].

The role of AtoSC, however, appears not to be limited to the regulation of the *atoDAEB *operon expression. Transcriptomic and phenotypic analyses of all two-component systems (TCSs) in *E. coli *implied communication among several systems [6]. In particular, deletion of *atoSC *genes resulted in a drastic alteration of the mRNA profiles, reduced motility, salt sensitivity and susceptibility to certain environmental agents. Despite the documented *in vitro *cross-regulation between the *E. coli *TCSs [7], the observation of such phenotypes implies AtoSC specific effects. These data suggest that other gene targets of the AtoSC TCS might exist and their identification could shed further light into the role(s) of this system.

Several approaches allow the prediction of transcription factor binding sites (TFBS). The most common of these are based on already existing, experimentally verified gene targets of transcription factors, which contain *cis*-regulatory elements in their promoter regions. The DNA sequences upstream of the transcription start sites (TSS) of these targets are used as input to various mathematical algorithms for the reconstruction of motif models, as for instance Gibbs Sampling [8] and over-representation of oligonucleotides [9]. Typically, these motifs are mathematical representations of conserved sub-sequences, emerging from a noisy background. The idea is that conserved DNA patterns would be overrepresented in a group of transcriptionally related sequences compared to a group of unrelated ones. Thus, conserved DNA patterns of co-expressed genes are more likely to be biologically relevant and hence to represent actual *cis*-regulatory elements. While a common method to define sets of co-expressed genes is transcriptomic analysis [10], the incorporation of phylogenetic information in the form of intergenic sequences upstream of orthologous genes can further enhance the accuracy of the motif detection. This is due to observations that regions playing an important role in gene regulation are more likely to be conserved even among different species [11-13]. Other methodologies use pathway or gene function information to assess co-regulation [14-16].

The common element in the aforementioned approaches is the assumption of prior knowledge of an adequate population of TFBS or the plethora of available co-regulation data. Neither of these two approaches could be used, however, for the prediction of the AtoC binding sites, since only a single AtoC binding site has been detected and the transcriptomic data are limited. For these reasons we set out to predict and then to detect AtoC binding sites through the setup of an iterative, genome-wide, *ab initio *detection method for the specific transcription factor, without the requirement of a population of target promoters or co-regulation data. The aims were the *in silico *prediction of additional binding sites, targeted by the AtoC response regulator and the experimental validation of these sites *in vivo*, using the ChIP technique.

## Results

### *Ab initio *motif detection and evaluation procedure

**Figure 1:**
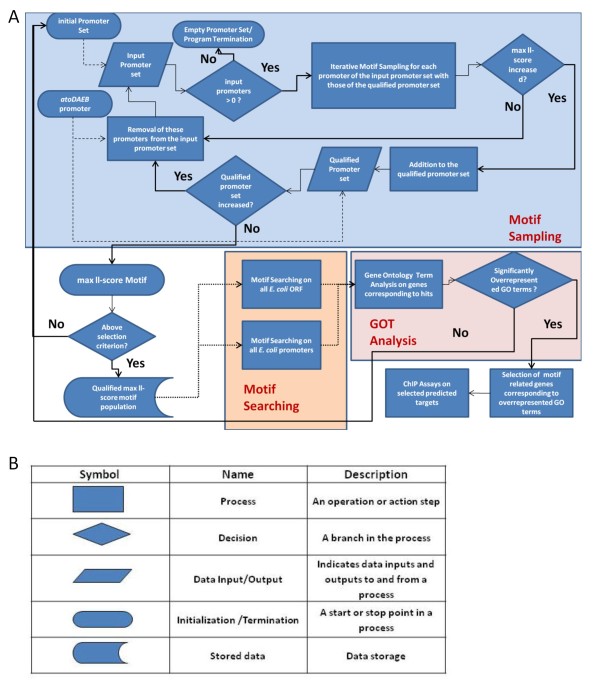
**Workflow chart of the *ab initio *motif detection procedure**. A) The chart describes the algorithmic steps for the *ab initio *motif detection process. The thick arrows correspond to algorithmic flows after decision points, the dashed arrows represent initialization steps which are performed uniquely and the dotted arrows (Motif Searching part) concern independently implemented alternative data processing scenarios. The details of the procedure are described in Materials and Methods. B) Description of the workflow symbols.

In order to predict putative AtoC binding sites, we implemented an *ab initio *motif detection procedure (Figure 1). This procedure involved an iterative pair-wise motif sampling process between the promoter of the *atoDAEB *operon, which was used as the initial qualified sequence, and a pool of *E. coli *promoters (initial promoter set). The promoter that showed the best homology to the *atoDAEB *promoter sequences was removed from the promoter pool and, together with the initial qualified promoter sequence, formed the qualified promoter set that was used for the next iteration of the motif-sampling process, against an input promoter set. In each iteration, the input promoter set is depleted by the selected promoter, plus those promoter sequences that scored below a predefined threshold (see Materials and Methods). The goal of this procedure was to yield motifs with maximized log-likelihood (ll) scores at the specified positions, thereby revealing conserved intergenic sites corresponding to putative AtoC binding sites. The ll-score represents an informative index of both the level of motif conservation (information content) and the number of its occurrences on a set of sequences. Promoters containing sequences, which yield motifs with high ll-scores, are pooled to form the final qualified set of promoters, which is used for the derivation of a best-scoring consensus motif.

Due to the fact that the MotifSampler program [17], which was used in this analysis, implements a heuristic algorithm, the sampling results are very sensitive to the input parameters. In particular, different motifs are obtained at the end of the procedure by slightly varying the parameters, e.g. the motif length, the motif maximizing positions or the initial promoter set (see Material and Methods). All these motifs represent different instances of sequence conservation between promoters. However, the sequence conservation among promoter regions is relatively small and this implies that other factors, e.g. the chromatin context or the formation of other transcription factor complexes, also contribute to the effective AtoC binding. Thus, among different sets of conserved sequences only a few would be biologically relevant. To cope with this issue and capture the highest possible number of biologically relevant motifs, the computational pipeline was applied many times with slight variations regarding the input parameters. Subsequently the biological relevance of the obtained motifs was assessed by implementing a Gene Ontology Term statistical analysis (see Materials and Methods).

Three executions of the procedure generated motifs that successfully passed the GOT test and were correlated with gene functions (Table 1). The first execution was initiated by pair-wise motif sampling the -184 to -165 region of the *atoDAEB *promoter, strictly containing the half AtoC binding sequence, against all *E. coli *promoters (initial promoter set, see Materials and Methods). The second execution also used the strict positions of the AtoD binding site but with the initial promoter set narrowed to only the sigma-54 dependent promoters from the RegulonDB database [18] because the *atoDAEB *promoter is itself sigma-54-dependent. The third execution was performed using another narrowed initial promoter set, consisting of the 57 promoters which are annotated in the *E. coli *K-12 EcoCyc database [19] as targets of response regulators of other TCSs. The three motifs derived from each execution are presented in Table 2. Besides these three successful executions, other attempts resulted to motifs that failed to yield GOT over-representation and they were hence discarded (data not shown), with the exception of motif 4 (see below). All retained motifs are provided in Additional File 1.

**Table 1:** The three executions of the motif detection procedure

Executions	Specified positions upstream TSS	Initial promoter set	Qualified promoter set
1	from -184 to -165	All *E. coli *promoters	atoD, galU, yhjR-bcsE, narZ, puuP, mdlA, dmsA, crr

2	from -184 to -165	sigma-54 factor-related promoters	atoD, rtcR, ibpB

3	from -184 to -165	Two-component target promoters	atoD, dmsA, borD, csgD, pstS, glnH, dctA, yebE, nirB, torC, asr, tppB, acrD,

The computational workflow was performed many times varying different parameters. Here are shown the three successful executions that corresponded to three different initial promoter sets.

**Table 2:** The four different motifs matching the palindromic AtoC binding site

Motif No.	Log-likelihood score	Consensus	Logo
Motif 1	117.6	GCkATrCrrnnATTTrCwCA	

Motif 2	51.8	GnGnAAnTTyCTGCAwAGCC	

Motif 3	168.6	wnnnwrsAwAwmrnknmAnr	

Motif 4	66.3	nCknATnmGrnnnTnACGCm	

Motifs 1-3 are the top scoring motifs derived of each of the three aforementioned executions. Motif 4 is an intermediary motif of unexpectedly high conservation among different intergenic regions.

### Genome-wide motif searching using the motifs obtained by the iterative procedure coupled to the Hypergeometric Test for Gene Ontology Term overrepresentation correlate motifs with gene functions

The biological relevance of the derived motifs was established by an algorithmic procedure, where each motif is used as input for genome-wide screening (Motif Searching) and the genes found to contain matching sequences within their promoter regions are tested through the Hypergeometric Test for Gene Ontology Term overrepresentation. Thus, if overrepresentation of a particular GOT is observed among the genes to which the motif hits were attributed, there is statistical evidence that this gene set was not randomly generated. As the permutation-derived corrected p-values impose a significant strictness into the statistical procedure, and since p-value correction discards the majority of GOT, which lack sufficient representation among the input genes, larger (more permissive) hit populations were required to ensure good statistical representation of all possible outcomes. Thus, the results of the Motif Searching algorithm significantly enriched the hit population (Table 3), since the qualified promoter sets were too small to yield GOT overrepresentation. In this way, the three aforementioned motifs were correlated to gene functions (Table 4) and they were selected for experimental validation. It is worth noting here, that not all these hits can map effective, functional binding sites under all experimental conditions; they rather represent putative targets, based on their sequence content. The statistical correlation with biological functions reveals slightly conserved sequences that are potentially inclined toward interaction with particular transcription factors. The GOT analysis, as implemented in this work, evaluates whether the information content captured by a motif implies random noise or is a biologically relevant one.

**Table 3:** Number of hits per motif at 0.7 prior probability

Motif	Hits (promoters)	Hits (ORFs
1	55	234

2	153	694

3	331	688

4	346	266

While motifs 1-3 matched more ORFs than intergenic sites, motif 4 matched an uncommonly conserved intergenic region.

**Table 4:** Results of the Gene Ontology Term Analysis

GO id	Term	Corrected p-value	FDR (%)	Enrichment in hits	Percentage in hits	Enrichment in genome	Percentage in genome
**Motif 1**							

GO:0016638	oxidoreductase activity, acting on the CH-NH2 group of donors	0.066	12.00	3/67	4.47	10/4339	0.23

**Motif 2**							

GO:0015749	monosaccharide transport	0.045	10.00	4/154	2.59	891101	0.23

GO:0022891	substrate-specific transmembrane transporter activity	0.006	0.00	22/154	14.28	254/4339	5.85

GO:0043169	cation binding	0.012	0.00	37/154	24.02	570/4339	13.13

GO:0008324	cation transmembrane transporter activity	0.076	2.33	13/154	8.44	128/4339	2.94

**Motif 3**							

GO:0045449	regulation of transcription	0.001	0.00	53/324	16.35	372/4339	8.57

GO:0007155	cell adhesion	0.027	0.00	10/324	3.08	34/4339	0.78

GO:0001101	response to acid	0.042	0.12	4/324	1.23	5/4339	0.11

GO:0009289	fimbrium	0.032	0.00	8/324	2.46	28/4339	0.64

GO:0003700	transcription factor activity	0.01	0.00	32/324	9.87	208/4339	4.79

**Motif 4**							

GO:0051234	establishment of localization	7.82E-04	0.00	40/110	36	800/4339	18

GO:0005215	transporter activity	3.91E-03	0.00	30/110	27	561/4339	13

Results of the GOT analysis of motifs 1, 2, 3 and 4, at prior probability set to 0.7 (corrected p-value cut-off set to 0.1).

### Evaluation of lower-scoring motifs and retention of Motif 4

In addition to the highest-scoring motifs, we also investigated motifs with intermediary correspondence to the AtoC-binding site. These motifs were initially discarded from the automatic procedure due to the stringent parameterization to retain only the highest-scoring motifs for the specified position. In order to overcome this limitation and test the discovery strengths of the proposed analytic pipeline in this work, we applied this to motifs that score lower during the Motif Sampling phase (assuming that they reveal weak matching with the *atoDAEB *promoter binding site) and examined them separately regarding their evaluation in the GOT analysis. In general, the hits of intermediary motifs either overlapped those of the highest-scoring motifs or did not provide any overrepresented GOT and so they were discarded (data not shown). Motif 4, however, is an exception. This intermediary motif was retained (Table 2) because it matched an unexpectedly highly conserved repeat. This particular repeat corresponds to an uncommonly conserved intergenic site present in many intergenic regions and yielded a significant GOT overrepresentation, when submitted individually to the motif evaluation sub-procedure.

The enrichment of motif 4 is particularly high within its hits (at 0.7 prior probability motif 4 comprises 346 hits on promoter regions; data provided in Additional File 2) due to the very high sequence conservation, and despite its restrictive character, which yields a lower log-likelihood score (66.3). Therefore, motif 4 was strongly correlated to the GOT Transporter Activity function (Table 4).

### Motif 4 resides within a ~40-bp highly conserved repeat found in many *E. coli *promoters

**Figure 2:**
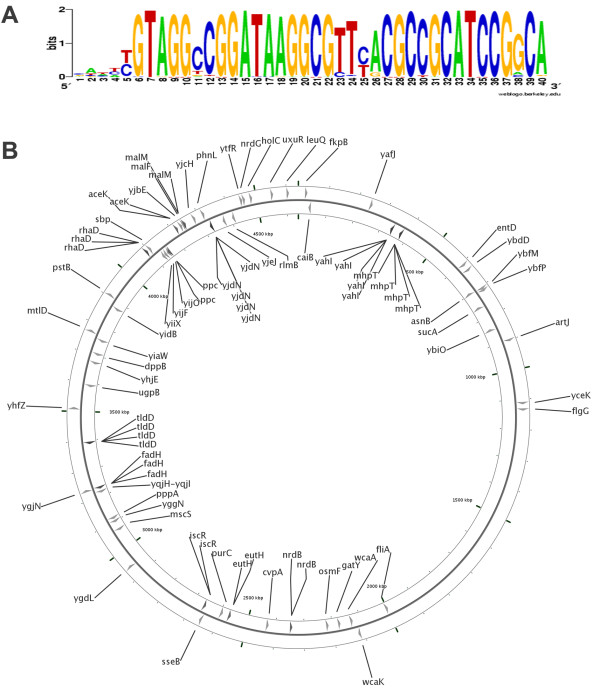
**Motif 5**. A) The sequence represents the highly conserved site of ~40 bp (based on 85 hits at 0.7 prior probability). B) The positions in the *E. coli *genome of the 85 hits of motif 5. All hits are situated in intergenic regions. The gene names correspond to the genes downstream of these intergenic regions. The arrow directions indicate the strand of each hit (to the right: strand +1; to the left: strand -1).

**Figure 3:**
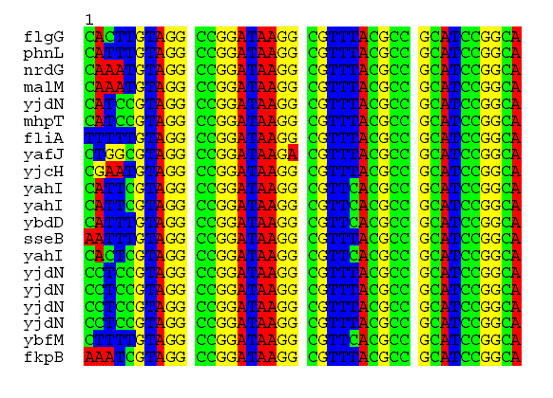
**Alignment of 20 top hits of motif 5**. The alignment of 20 top hits of motif 5 highlights the high sequence conservation of a ~40 base pair-site located exclusively in intergenic regions (85 in total) and may have a putative global regulatory role in *E. coli *and other *Enterobacteria*.

Motif 4 weakly matched the palindromic AtoC binding site in the promoter of the *atoDAEB *operon [5] and at the same time it strongly matches an unexpectedly conserved ~40-bp repeat present in a significant number of promoters (motif 4 searching results, Additional File 2). An additional motif, motif 5, was constructed in order to represent this conserved region. The sequence of motif 5 at 0.7 prior probability is shown in Figure 2A. Motif 5 was used for genome-wide screening and matched 85 intergenic sites, whereas it did not match any site within ORFs (data provided in Additional File 2). The high degree of conservation of this 40 bp repeat and its wide representation in the genome (Figure 2B) suggests a more pivotal role in global *E. coli *regulatory mechanisms. The alignment of the first twenty hits shows unexpected sequence conservation, for an intergenic region (Figure 3) where, for example, there are 6 variants of the repeat with at least 38/40 identity in various intergenic positions. The probability of finding a 38-bp DNA sequence repeated by chance two times is 1/0.25^38 ^× 4 in the *E. coli *genome comprising 4.6 × 10^6 ^nucleotides. This result is even more pronounced if we consider the absolute establishment of the repeat only within intergenic regions and not within ORFs.

Interestingly, this highly conserved sequence was also detected in many intergenic regions of other *Enterobacteria *such as *Shigella flexneri, Salmonella enterica, Klebsiella pneumonia *and *Citrobacter coseri *(data not shown).

### Application of the computational procedure on the transcription factor LexA as a demonstration of its general effectiveness

In order to demonstrate that the computational procedure used here for the prediction of AtoC binding elements can be applied to other transcription factors, we analyzed its effectiveness using LexA, a well-characterized transcription factor. LexA, which regulates the transcription of several genes involved in the cellular response (SOS response) to DNA damage or inhibition of DNA [20], has been extensively studied and the multitude of experimentally identified DNA binding sites that predicted its consensus [21] were also used as a benchmark to assess the predictive capacity of our computational framework. By initiating the computational analysis with one LexA binding sequence and querying the whole promoter set of *Escherichia coli *K12 (motif length 20, same parameters as for AtoC), we identified a number of putative DNA binding sites, including thirteen known LexA binding sites. Moreover, the highest-scoring GOTs generated from this procedure were GO:0006974, "response to DNA damage stimulus" and GO:0009432, "SOS response", a finding that is in total agreement with the established biological function of LexA (the findings are presented in Additional File 3).

### Experimental validation of the *in silico *predicted sequences by *in vivo *binding of recombinant AtoC

ChIP experiments are routinely used to define whether a DNA-binding protein recognizes a particular gene regulatory region *in vivo*. Regarding the system under study, the specificity of the results obtained with this method has already been demonstrated by the lack of AtoC binding to its defined site in the regulatory region of the *atoDAEB *operon in the *E. coli *strain BW28878, an *atoSC *deletion mutant [6]. In contrast, in the isogenic *atoSC^+ ^*strain BW25113 AtoC was found to bind this site *in vivo *and this binding was more pronounced in the presence of inducing acetoacetate [5]. Having confirmed that AtoC binds *in vivo *to its *a priori *target, ChIP experiments were used to define whether AtoC binds to any of the putative targets that emerged from the bioinformatics approach. Initially, we analyzed potential AtoC binding to four such sequences, three located within the *dmsA, acr *and *fliA *promoter regions, and one located within the *fliT *gene-encoding region. ChIP experiments indicated that AtoC binds to all four, as well as in the control *atoDAEB *promoter (data not shown). The specificity of the experimental approach used was confirmed by the lack of any visible signal, when the precipitations were performed either in the absence of AtoC-specific antibody or in the *atoSC¯ *strain BW28878.

**Figure 4:**
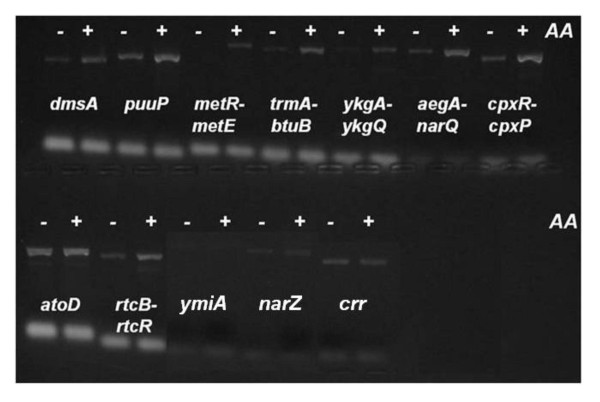
***In vivo *binding of recombinant AtoC to *in silico *predicted potential targets of motif 1 in intergenic regions**. Results of a ChIP analysis by agarose gel electrophoresis (2% w/v) of PCR amplification products generated by primers corresponding to motif 1 hits and qualified promoters, included in Tables 1 and 5. All ChIP preparations were carried out following acetoacetate induction. Above each PCR product, generated by the specific designated primers, the name of each gene or promoter region comprising the motif sequence is denoted for each pair of lanes. All AtoC additional binding targets are PCR tested (25 cycles) in two ChIP preparations carried out without (-, left lanes) or with (+, right lanes) AcAc induction (*AA*).

**Figure 5:**
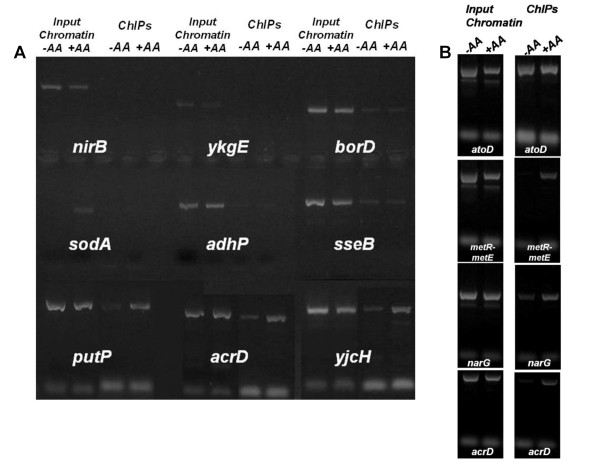
**Monitoring the AcAc effect on AtoC *in vivo *binding to its predicted "atypical" targets**. A) All predicted targets matching motifs 2, 3 and 4 (Table 5), that gave a positive signal in genomic DNA, are ChIP analysed. All AtoC additional binding targets are PCR tested (25 cycles) in two ChIP preparations carried out without (-, left lanes) or with (+, right lanes) AcAc induction (*AA*). Input Chromatin samples are also included representing positive genomic DNA signals for the sampled targets. B) Three selected targets representing each of the afore-mentioned groups (*metR-metE, narG *and *acrD*) were also PCR tested together with *atoD *(25 cycles) using as templates input chromatin controls (Input Chromatin) and immunoprecipitated preparations (ChIPs) from non-induced (*-AA*) or AcAc induced (*+AA*) cultures.

To further increase the probability that AtoC would bind *in vivo *to even relatively low affinity targets predicted by the bioinformatic analysis, the experiments were performed in *E. coli *BL21[DE3] transformed with plasmid pHis_10_-AtoC [22] and overexpressing a recombinant His-tagged form of AtoC, recognised by an anti-His probe. Initially, we analyzed potential AtoC binding to the hits of the qualified promoter sets and subsequently to additional hits of the motif derived from each execution. Ten of the qualified promoters of Table 1 were tested and found positive for *in vivo *AtoC binding, together with the already verified *atoD*: *narZ *[23], *puuP *[24], *dmsA *[25], *crr *[26], *rtcB-rtcR *[27], *borD *[28] and *acrD *[29]. ChIP analysis was also extended to accommodate targets derived from the Motif-Searching procedure while their cognate gene products suggested biological relevance (presented in Table 5). Sequences within six intergenic regions, i.e. *metR*-*metE *[30], *trmA-btuB *[31], *ykgA-ykgQ *[32], *aegA*-*narQ *[33,34], *ymiA *[35], *narZ *[23], and *cpxR-cpxP *[36], which were observed as hits by motif 1 searching (Table 2) were selected for testing based both on their scores and their putative functional relevance to the *ato *genes. The results of the ChIP analysis for all intergenic regions ascribed to motif 1 are included in Figure 4, which clearly demonstrates the *in vivo *binding of recombinant AtoC. Six additional promoters, i.e. *borD *[28]*, putA*-*putP *[37]*, acrD *[29]*, adhP *[38], *rhaT*-*sodA *[39,40] and *nirB *[41], that are likely transcribed by sigma-54 polymerase and which matched motif 2, were similarly tested for *in vivo *AtoC binding. Finally, both the *borD *and *acrD *gene promoters, to which AtoC was found to bind *in vivo*, share patterns with motif 3. Regarding the top hits of motif 4 (Additional File 2), *sseB *and *yjcH *were also among the positive targets. This result is potentially significant since *yjcH *is cotranscribed with *acs *and *actP*, which encode proteins involved in acetate activation and transport, as part of the *acs-yjcHG *operon [42-44]. All predicted targets matching motifs 2, 3 and 4 that gave a positive signal in genomic DNA (Input Chromatins, see paragraph regarding specificity of AtoC binding) were also analysed by ChIP and the results are presented in Figure 5A. As becomes evident in this figure, not all targets are recognised by AtoC with the same intensity, even though the input chromatin signals share, as stated above, the same strong level of intensity. This allows a certain prioritization of the putative targets according to the strength of the AtoC binding.

**Table 5:** Sites experimentally tested for *in vivo *AtoC binding

Result	Site	Gene	Motif	Strand	Site distance from TSS (Refseq)		Gene product
+	GCTATGCAGAAATTTGCACA	atoD	1,2,3,4	(+)	-184	-165	acetyl-CoA: acetoacetyl-CoA transferase, subunit

+	GCTATGCAGAAAATTGCGCA	atoD	1,2,4	(-)	-163	-144	acetyl-CoA: acetoacetyl-CoA transferase, subunit

+	GCTATAGAAATAATTACACA	dmsA	1,2	(-)	-69	-50	dimethyl sulfoxide reductase, anaerobic, subunit A [25]

+	GCCATGCGGGGATTTAATCA	puuP	1	(+)	-148	-129	putrescine importer [24]

+	GCCGTCCAGATGTTTACACA*	metR-metE	1	(-)	-87, -170	-68, -151	transcriptional activator [30]

+	GCGATTGTAGGGATTGCTCA*	trmA-btuB	1,(2)	(+)	-49, -340	-30, -321	tRNA (uracil-5-)-methyltransferase/ outer membrane transporter [31]

+	ACTATACGGAAAATTCCACT*	ykgA-ykgQ	1,2	(-)	-235, -55	-216, -36	Putative transcriptional activator [32]

+	GTGATGGGATTATTTGATCT*	aegA-narQ	1	(-)	-147, -79	-128, -60	predicted fused oxidoreductase: FeS binding subunit and sensory HK in TCS with NarP [33,34]

+	GCCTCGGAGGTATTTAAACA*	cpxR-cpxP	1	(-)	-21, -149	-2, -130	response regulator in TCS with CpxA/ periplasmic protein; combats stress [36]

+	GCGAGGCGGGTAATTAGACA	ymiA	1	(-)	-199	-180	small predicted membrane protein [35]

+	GGCCCAATTTACTGCTTAGG	crr	1,2	(+)	-28	-9	glucose-specific PTS component [26]

+	GCGGTGCAGGAGATTGCACA	fliT	1	(+)	127	146	predicted chaperone (flagellum) [46]

+	CCGGTCCAGGAATTTACTCA	narG	1	(-)	2	21	nitrate reductase 1, alpha subunit [23]

+	GCGAAACGATTAATTACACA	ycjM	1	(+)	94	113	glucosyltransferase predicted [64]

+	ACTGTACAGAAAGTTGCTCA	yeaM	1	(-)	668	687	transcription regulator (predicted) [65]

+	GCGGGTCAGGTAATTGCACA	gadA	1	(-)	959	978	glutamate decarboxylase A, PLP-dependent [66]

+	GACATGCAGTTGATTACACA	eutR	1	(+)	783	802	predicted transcription regulator [67]

+	GCGATCCAAAAGTTTACTCA	narZ	1	(+)	0	+ 19	nitrate reductase 2 alpha subunit [23]

+	CGGAGATTTCCCGCAAAGCC*	rtcB-rtcR	2	(+)	-145, -64	-126, -45	sigma-54-dependent transcriptional regulator of rtcBA [27]

+	CGGCAAGTTTCGACATTGCC*	putA-putP	2	(+)	-398, -45	-379, -26	fused transcriptional regulator/proline dehydrogenase/ carboxproline:sodium symporter [37]

-	GGGCATTTTCCTGCAAAACC*	rhaT-sodA	3	(+)	-167, -138	-148, -119	L-rhamnose:proton symporter/superoxide dismutase [39,40]

-	GGATGATGTTCTGCATAGCA	adhP	3	(+)	-35	-16	ethanol-active dehydrogenase/acetaldehyde-active reductase [38]

-	TTTATAGAAATAGATGCACG	nirB	4	(-)	-205	-186	nitrite reductase, large subunit, NAD(P)H-binding [41]

+	TATGTGTAGAAAATTAAACA	borD	4,3	(+)	-59	-40	DLP12 prophage; predicted lipoprotein [28]

+	GTTCAGTATAAAAGGGCATG	acrD	2	(+)	-128	-109	aminoglycoside/multidrug efflux system [29]

-	GAAATAGAAATAGTTGAAAG	ykgE	4	(-)	-472	-453	predicted oxidoreductase [32]

+	CCGGATAAGGCGTTTACGCC	yjcH	4	(+),(-)	-114, -122	-95, -103	conserved inner membrane protein/ acetate transport [44]

-	CCGGATAAGACGTTTACGCC	yafJ	4	(+)	-193	-174	Unknown function, similarity to type II amidotransfease

+	CCGGATAAGGCGTTTACGCC	sseB	4	(+)	-125	-106	rhodanese-like enzyme [68]

Motif hits experimentally tested by ChIP analysis for *in vivo *AtoC binding. All annotations were provided by the Refseq database (*Escherichia coli *K12, Refseq id: NC_000913). Underlined: hits in gene-encoding regions. *These hits represent a unique site per pair, which could be attributed as *cis*-regulatory element to both flanking genes. As predicted by the GO Term analysis many of the targets are transcription factors, transporters and enzymes involved in oxidoreduction.

### *In vivo *binding of AtoC to gene encoding regions

**Figure 6:**
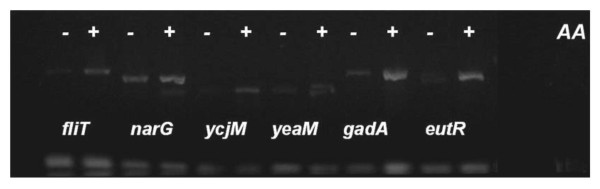
**Detection of putative AtoC binding targets in gene encoding regions**. Selected motif 1 hits in gene encoding regions (Table 5) were ChIP analyzed for *in vivo *AtoC binding by electrophoresis of PCR products generated by primers specifically designed to comprise the target motif sequence. The name of each gene comprising the motif sequence is denoted above each lane. The AcAc effect on the binding to each target is also denoted, as stated in Figure 4.

An interesting aspect of this bioinformatic analysis is the detection of several potential AtoC binding sites, corresponding to the pattern of motif 1, within gene-encoding regions. The hits of motif 1 screening within ORFs are shown in Additional File 2. Seven of them could have functional relevance to the *ato *system based on their GOT analysis. ChIP experiments were performed to identify whether AtoC binds to them *in vivo *and the results of this analysis were positive for six out of the seven gene-encoding regions tested, i.e. *fliT, narG, ycjM, yeaM, gadA *and *eutR *(Figure 6). Thus, it is most likely that AtoC also binds within the gene-encoding regions predicted by the bioinformatic analysis, although the functional relevance of this binding remains unclear.

### Specificity of AtoC binding to its predicted targets: the effect of the acetoacetate inducer *in vivo *and *in vitro *DNA-binding competition experiments

In order to assess the specificity of AtoC binding to the predicted operators, we re-examined all ChIP analyses by reducing the PCR cycles to twenty five, while ChIP signals for both acetoacetate-induced and non-induced cultures were analysed comparatively for each target. AtoC was found to demonstrate increased affinity for most of the predicted "atypical" sites only in the case where acetoacetate, the inducer, was added to the cultures, while the abundant, over-expressed AtoC appears able to bind the *atoDAEB *promoter with the same affinity, regardless of the presence of acetoacetate (Figures 4, 5 and 6). In an attempt to ensure consistency of the results through the use of extra controls, chromatin input samples for all targets groups were also tested for PCR positive signals in the absence of the His-probe antibody precipitation. DNA signals of the chromatin input for both induced and non-induced cultures, exhibited the same signal intensity (Figure 5B), which proves that the signal intensity corresponding to each target can be solely attributed to the AtoC binding, whereas the absence of signal can be considered a sign of absence or weak binding, rather than a mere coincidence caused by primer malfunction. Moreover, low ChIP signals for the non-induced cultures provide negative controls, in the presence of both abundant AtoC and the antibody, directly relating the presence of abundant positive signals to acetoacetate induced AtoC binding of the predicted targets, thus supporting our case.

**Figure 7:**
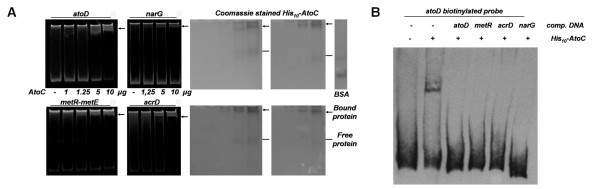
**Verification of AtoC *in vitro *binding to representative "atypical" targets**. A) Sample gel retardation experiments illustrating His_10_-AtoC protein binding to DNA fragments *atoD, metR-metE, acrD *(promoter) and *narG *(gene encoding) regions. As described in Materials and Methods, following electrophoresis, gels were first stained with Gel-Red dye and then with Coomassie blue, and side-by-side photographs are shown. The lines above the Gel-Red stained lanes indicate the different probes combined with the indicated His_10_-AtoC (AtoC) quantities. Arrows indicate bands that were stained with both Gel-Red and Coomassie blue, corresponding to band-shifting caused by the AtoC binding. Lines indicate free proteins, not bound to corresponding DNA particles. B) Band shift assay of His_10_-AtoC with a biotinylated fragment of the upstream region of the *atoDAEB *operon. EMSAs were performed as described in Materials and Methods with 10 ng of biotinylated *atoD*. The addition of His_10_-AtoC (0.35 μM) and certain amounts of competitive, non-biotinylated *atoD*, as well as *metR-metE, acrD *and *narG *fragments is indicated. 2 μg of sonicated calf thymus competitive, non-specific DNA were added in each reaction.

To further address whether AtoC indeed binds to the predicted targets, the aforementioned representative of each of the three groups was also tested, together with *atoD*, in gel retardation assays combining His_10_-AtoC with the PCR products that contain the predicted promoters or gene encoding regions. The specificity of the DNA-AtoC interactions has already been verified by competition experiments to evaluate the association of AtoC with biotinylated *atoD *probe in the presence of increasing concentrations of unlabeled specific (*atoD*) or nonspecific DNA competitors [4,5]. As illustrated in Figure 7A, electrophoretic gel retardation assays showed that all DNA targets exhibit binding to AtoC, following incubation on ice for 25 min. However, this binding appears to be less pronounced when compared with the cognate, high-affinity AtoC binding site present in the *atoD *promoter. The specificity of the results was further verified by also performing DNA binding reactions on ice for 30 min in the presence and absence of DNA competitors as previously described [4,5]. Under these conditions, His_10_-AtoC binding to the biotin-labelled *atoD *probe was abolished either by the addition of excess amounts of unlabelled cognate (*atoD*) competitor or by unlabelled DNA competitors containing the novel, atypical binding sites, i.e. *metR-metE, acrD, narG *(Figure 7B). The degree of specificity of the AtoC binding to each of the predicted targets remains to be determined.

## Discussion

**Figure 8:**
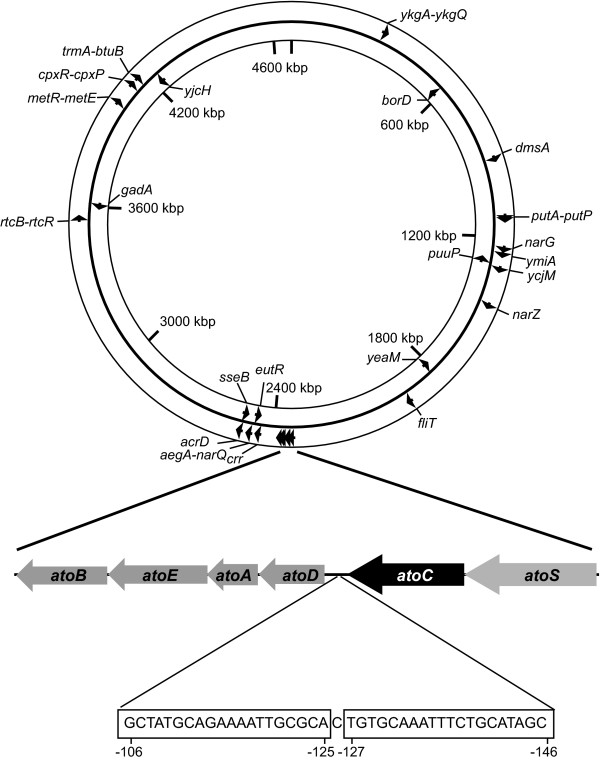
**Location of *atoS*-*atoC, atoDAEB *operon genes relative to the positions of the verified AtoC targets on the *E. coli *genomic map**. The positions of the additional twenty-two experimentally verified AtoC binding sites, are shown on the circular *E. coli *genomic map, with the arrows indicating the direction of transcription. The *atoSC *and *atoDAEB *regions are expanded and the sequence of the inverted palindromic repeat, representing the AtoC original target, is shown in the boxes.

The AtoSC two-component signal transduction system is involved in the expression of the *atoDAEB *operon encoding proteins required for the catabolism of short-chain fatty acids. Acetoacetate activates the AtoSC TCS, and subsequently, the expression of the *atoDAEB *operon genes. We have already shown that, upon acetoacetate activation, AtoC binds a 40-bp palindromic sequence (two identical inverted 20-bp sites) located upstream of the *atoDAEB *transcription start site and activates the sigma-54-dependent expression of this operon [5]. A schematic representation of the *atoSC *and *atoDAEB *region, as a well as the positions of the verified AtoC targets including the high-affinity binding site, on the *E. coli *genomic map, is illustrated in Figure 8. In addition to its role as a transcriptional activator, AtoC has a second function as the posttranslational inhibitor of ornithine decarboxylase, the key-enzyme in polyamine biosynthesis [45]. These data underline the complexity of the regulatory networks that are involved in maintaining the metabolic pathways and homeostasis in bacteria.

Several lines of evidence indicate that the role of the AtoSC TCS in the regulation of *E. coli *gene expression might not be limited to the expression of the *atoDAEB *operon genes. Deletion of the *atoSC *locus results in the inability of *E. coli *to catabolize short-chain fatty acids, as well as in a number of other defects including down-regulation of flagellar gene expression, defects in motility and chemotaxis, inability to use glucuronamide as carbon source and sensitivity both to high osmotic pressure and to aminoglycoside antibiotics [6]. These pleiotropic effects could involve the phosphorylation of non-cognate response regulators by the AtoS kinase and the regulation of the expression of additional genes by AtoC, as part of a cross-talk regulatory network. This study exposed a number of promoters of related genes that may serve as putative AtoC targets. These include *fliT *that encodes a chaperone of the flagellar export system [46], and the neighboring *fliA *gene that encodes the sigma-28 polymerase, which activates transcription of both flagellar and chemotaxis genes, including *tar, tsr *and the *flaA *operon [47-49]. Additional potential AtoC targets include *acrD*, which encodes a component of the AcrAD-TolC multidrug efflux transport system and is involved in bacterial response to a variety of aminoglycosides [29], *puuP *encoding an importer for putrescine [24], the enzymatic product of ornithine decarboxylase and the precursor of the polyamines spermine and spermidine [19] as well as *yjcH *encoding a conserved inner membrane protein involved in acetate transport [44].

A defined consensus sequence for the AtoC binding site would greatly facilitate the identification of potential AtoC binding elements. However, the lack of experimentally verified AtoC binding targets other than that upstream of the *atoDAEB *promoter [5], necessitates the utilization of bioinformatics methodologies for the task of predicting potential AtoC binding motifs. Therefore, we implemented an *ab initio *computational prediction of additional targets, using half the verified palindromic sequence of the AtoC binding site [5] as the sole input. The process, executed many times with slightly varying parameters, generated different motifs, which were tested through statistical GOT enrichment analysis for their biological relevance. The qualified motifs defined a pool of putative AtoC binding sites present within either intergenic or gene-encoding regions of the *E. coli *genome. The biological validation of the *in silico*-obtained predictions was performed by ChIP assays that demonstrate AtoC binding *in vivo*. These experiments validated the computational approach, since AtoC was found to bind *in vivo *in twenty-one of the predicted target sequences. As predicted by the GOT analysis, many of the targets are transcription factors, transporters and enzymes effecting cellular redox state (Table 5).

As no high homology is shared among the primary sequences of the confirmed AtoC binding sites, the extrapolation of a strict consensus sequence remains elusive. Previous studies have shown that global bacterial transcription factors can recognize multiple non-canonical binding sites that do not conform to a consensus site [50,51]. Our computational workflow bypassed this issue by revealing slightly conserved, yet functionally effective, AtoC binding sites that do not conform to a global consensus. Another interesting aspect of this analysis is that AtoC binds *in vivo *not only to intergenic sequences but also to a number of gene-encoding regions. It is not clear whether this binding has a regulatory (possibly enhancing) impact on the expression of these genes or whether it serves some other purpose, such as quenching the amounts of available AtoC within the *E. coli *cell milieu. AtoC, like other response regulators, could in theory affect bacterial gene expression from a distance [52], in a manner analogous to that of eukaryotic enhancer-binding transcription factors [53]. However, this is not necessarily the case, since there is at least one report of response regulator binding to gene-encoding regions without obvious effects on gene expression [54]. The presence of alternative transcriptional factor DNA targets of unknown function has been recently demonstrated [55], showing that CtrA, a bacterial response regulator, binds a second category of weak DNA targets. Questions remain regarding the conditions governing the AtoC binding to these sites *in vivo*, i.e. whether AtoC binds alone or in conjunction with some other factor(s).

There is a high probability that existing public microarray data cannot reveal the full extent of the biological relevance of the AtoSC system, since those data were obtained from cells grown in very rich media. Our data clearly indicate that conditions promoting the activation of the AtoSC TCS, i.e. the presence of the acetoacetate inducer, are required to promote AtoC binding to the predicted sequences. In this sense, microarray experiments from cells exposed to AtoSC inducers, and the subsequent correlation of these data with the novel AtoC binding targets presented here, could provide significant information on the biological importance of the AtoSC TCS.

Furthermore, the wealth of novel AtoC-binding sites, some of which are present within gene-encoding sequences, should be further analyzed for their functional significance in gene regulation. While this may not be a trivial task, especially when taking into account the large number of targets, it should provide insight into the regulation of bacterial gene expression from a distance.

## Conclusions

The scope of this work was to employ an *ab initio *motif detection approach, coupled with gene ontology analysis, in order to define novel binding elements for the bacterial transcription factor AtoC. This approach identified a number of sequences which, albeit their divergence, were recognized by AtoC *in vivo*. Overall, this study underlines the emergent complexity of a possible AtoC-related regulatory network which may contribute to the pleiotropic effects of AtoC.

## Materials and methods

### Computational Tools

All procedures were implemented on a Linux workstation using a combination of Perl scripts interacting with the specified INCLUSIVE programs for motif analysis (MotifSampler, MotifSearch, MotifRanking) [17]. All sequences, annotations and intermediary results were stored and queried in a Mysql database using the Perl-Mysql interface DBI library.

### Selection of promoters

The promoters are defined here as the sequences from -250 to +30 relative to each transcription start site (TSS). The annotations of the TSS and the genomic sequence of *Escherichia coli *K-12 were extracted from the Refseq database [56] (refseq id:NC_000913) using Perl and Bioperl scripts. No overlap between two promoters was allowed and all intergenic sequences shorter than 500 base pairs and between two genes encoded respectively in -1 and +1 strands were entirely included and considered as promoters of both flanking genes in the GO Term analysis step.

### Definition of promoter sets

The initial promoter set is defined as the promoter pool that is subjected to motif sampling. The different initial promoter sets used in the three executions of this analysis are presented in Table 1.

The promoters submitted to each reiteration of motif sampling represent the input promoter set. During the first iteration the input promoter set, which equals the initial promoter set, is subjected to motif sampling against *atoDAEB *promoter sequences representing the initial qualified promoter set. The number of promoters within the input promoter set decreases after each reiteration, since both a qualified promoter, as well as promoters scoring below a threshold value (see below), are eliminated from the pool. The qualified promoter set, ever increasing by one, is then used for the next iteration of the motif sampling process and the procedure advances until no more qualified promoters can be found.

### Iterative procedure for *de novo *motif detection

The *de novo *motif detection was performed using a Gibbs sampling algorithm (implemented in INCLUSIVE MotifSampler program [17]) in a multi-step procedure. The first step of the procedure was a pair-wise motif sampling between the *atoDAEB *operon promoter and each of the other *E. coli *promoters of the initial promoter set (3789 in total in the case of the full set of *E. coli *promoters), using the following parameters: A hundred runs (detection of one motif per run permitted); motif length 20; both strands; background model of order 3 (downloaded from the INCLUSIVE [17] web site for *E. coli *K-12), with prior probability value 0.5. The program retained the highest scoring motif.

In each iteration:

- A log likelihood (ll) score is estimated for every motif derived from the pair-wise motif sampling between each remaining promoter in the input promoter set and the qualified promoter set derived from the previous sampling iterations.

- The promoter of the input promoter set yielding a motif with the maximum ll-score within the specified position of the *atoDAEB *promoter is retained and added to the qualified promoter dataset. Thus, the qualified promoter set is derived through iterative agglomeration.

- The motif sampling performs independent rounds (same parameters as above) and datasets composed of n+1 sequences, where "n" the number of sequences in the dataset of the previous step (started by 2).

- When the ll score of a particular motif derived is lower than the 30th percentile of all scores, then this promoter is immediately eliminated from the input promoter set.

- Additionally when the position-specific maximum ll-score of a motif within a promoter is either equal or lower than the score derived in the previous step, then this promoter is also removed from the input promoter set.

In order to reduce the number of motif sampling rounds, the elimination of promoters that are less probable to maximize the ll-scores of the detected motifs is predicated, otherwise the screening procedure becomes time-exhaustive and possibly non converging to a final qualified promoter set, with an increasing number of sequences embedded in the qualified promoter dataset.

The procedure was successfully performed three times using all the same parameters except the initial promoter set. The first execution comprised the full set of all *E. coli *promoters. For the other two executions the initial promoter set was either the set of sigma-54-dependent promoters retrieved from the RegulonDB database [18] (*argT, astC, chaC, dcuD, ddpX, fdhF, glmY, glnA, glnH, glnK, gltI, hycA, hydA, hyfA, hypA, ibpB, kch, nac, norV, potF, prpB, pspA, pspG, puuP, rpoH, rtcB, rtcR, rutA, yaiS, ybhK, yeaG, yfhK, ygjG, yhdW, zraP, zraS*) or the set of promoters that comprise binding sites for other response regulators of two-component systems and are annotated in the *E. coli *K-12 database EcoCyc [19] as targets of response regulators (*dmsA, emrK, gadE, yca, spy, cydA, sucA, cpx, rdoA, ppiA, yqjA, acrD, ompC, mdtA, ftnB, yebE, ydeH, ycfS, efeU, yccA, aroG, ppiD, degP, ybaJ, cusR, dctA, frdA, dcuB, astC, kdpF, nrfA, nirB, hcp, napF, tppB, ompF, csgD, psiE, pstS, asr, phoH, phoB, phoA, phoE, mgtA, yrbL, mgrB, rstA, nagA, borD, hemL, ftsA, osmC, tnaA, torC, uhpT, zraS*). For all three executions, the positions from -184 to -165 upstream the *atoDAEB *operon were used as input parameter.

### Motif Ranking

The motifs generated by Motif Sampling were ranked and separated by Kullback-Leiber distance (INCLUSIVE *MotifRanking *program [17]). The threshold was set to 0.9 which means that if the Kullback-Leiber distance between two motifs is smaller than this threshold these motifs are considered as similar.

### Motif Evaluation and correlation with biological function

The evaluation of the motifs was performed by coupling Motif Searching with Gene Ontology Term Analysis. The motif searching hits were attributed to genes and these genes were submitted to the Hypergeometric Test for GO Test over-representation, described below.

#### • Motif Searching

The position-specific probability matrices (PSPM) representing these motifs, were used for two types of motif screening (INCLUSIVE MotifScanner program [17]); one including all individually cut intergenic sequences and another including all individually cut gene-encoding sequences. For both screenings the prior probability value was set to 0.7. The motif sequences were constructed by the WebLogo program [57]. For the sequence alignment we used the Seaview program [58].

#### • Gene Ontology Term Analysis of the promoters containing the motifs

In order to assess whether these motifs correspond to a *cis*-regulatory element, we performed the Hypergeometric Test for GO Test over-representation in the genes having a promoter, where a hit was observed at the Motif Searching step. For the implementation of the test, we used the GO::TermFinder Perl module [59]. The GO Term gene annotation used was the EBI Gene Ontology Annotation (GOA) [60] for *E. coli *K-12 (84.4% coverage). The p-value threshold of the Hypergeometric Test was set to 0.1 for the first test and to 0.05 for the second one (for corrected p-value, using 1000 simulation runs) and we considered as the total gene population the 4339 *E. coli *K-12 genes annotated with GO terms.

### Construction of motif 5

The promoters which contained hits of motif 4 were submitted to MotifSampler with the same parameters as the initial motif samplings, except from the motif length, which was set to 40. For the visualization of the genomic positions of the hits of motif 5 we used the CGview Server [61].

### Bacterial strains and growth conditions

Cells were grown to their mid-exponential phase at 37°C in 10 ml of modified M9 mineral medium supplemented with 0.1 mM CaCl_2_, 1 mM MgSO_4_, 0.4% (w/v) glucose, 1 μg of thiamine/ml and 80 μg of proline/ml [62], in the absence or presence of 10 mM acetoacetate. *E. coli *BL21[DE3] cells carrying pHis_10_-AtoC plasmid [22] were grown at 37°C in minimal medium containing 100 μg/ml ampicillin. Induction of recombinant AtoC expression was achieved by addition of IPTG (0.25 mM) to the cultures when the OD_600 _reached 0.25 and it was allowed to take place for 4 hours. Acetoacetate, the known inducer of the AtoSC TCS, was also added during the induction phase of the culture at a concentration of 10 mM.

### ChIP assays

ChIP assays were performed using chromatin from *E. coli *BL21 [DE3] over-expressing His-tagged AtoC, grown in the absence or presence of acetoacetate. The assays were performed using Magna ChIP™ A Millipore kit following the manufacturer's instructions. Briefly, *in vivo *cross-linking of the nucleoproteins took place by the addition of formaldehyde (final concentration 1%), directly to the *E. coli *(10 ml cultures) grown in modified M9 mineral medium, when the OD_600 _of the culture reached 0.6. The cross-linking reactions were quenched 20 min later by the addition of glycine and the cells were harvested by centrifugation and washed twice with ice-cold PBS, supplemented with a protease inhibitor cocktail. Cell lyses were performed as described in the kit manual and the cellular DNA was sheared by sonication to an average size of 500 to 1,000 bps. Cell debris was removed by centrifugation and the supernatant was used as the chromatin input for the immunoprecipitation reactions, after an initial stage of pre-incubation with protein A magnetic beads, in the absence of antibodies, to remove the nonspecific binding proteins. The ChIP reactions were then performed by adding the His-probe™ Santa Cruz rabbit polyclonal) and fresh protein A magnetic beads, to the resulting supernatant and allowing the reactions to take place overnight, with rotation at 4°C. DNA was then obtained from the immunoprecipitated protein/DNA complexes and the presence of the target promoter sequences in the chromatin immunoprecipitates, was detected by PCR amplification. Parallel mock precipitations performed in the absence of added antibody, were used as negative controls for each reaction.

### Primer design and PCR reactions

The primers used to monitor the chromatin immunoprecipitations (Additional file 4) were designed, using the Oligo Explorer software, to generate a product of approximately 300 base pairs. All oligonucleotides were synthesized by VBC Genomics, Austria. The number of PCR amplification cycles varied from 25 to 35 to achieve the maximum sensitivity and specificity. The PCR products were analyzed by electrophoresis on 2% w/v agarose gels and stained using GelRed (Biotium). DNA polymerase DyNAzyme™ EXT and reaction buffers used in the PCR process were purchased from Finnzymes. *Msp*I digest DNA ladder was purchased by New England Biolabs.

### Electrophoretic Mobility Shift Assays

We used the electrophoretic mobility shift assay methods of Orchard and May [63], slightly modified, to determine the formation of protein-DNA complexes. The His_10_-AtoC protein was bound to double-stranded oligonucleotides spanning the representative target sites of the three promoters (*atoD, metR-metE *and *acrD*) and the *narG *gene. Respective quantities of each of the four indicated particles were combined with elevated His_10_-AtoC quantities (1, 1.25, 5 and 10 μg) in 25 μl of binding buffer, 10 mM Tris [pH 7.6], 10 mM MgCl_2_, 50 mM NaCl, 5% (v/v) glycerol, and incubated on ice for 25 min, following a 5 min pre-incubation at room temperature, before the addition of protein. Non-protein containing samples were quickly mixed with 10× Gel loading buffer (Takara) and all samples were applied to a 100 V prerun polyacrylamide gel containing 0.5× TBE buffer prepared with 6.0% (w/v) polyacrylamide (ratio of acrylamide to bisacrylamide, 29:1. Following electrophoresis (120 V, 120 min), the gels were stained first with Gel-Red dye (Biotium) and then with Coomassie blue.

For the DNA-binding experiments 10 ng (3 nM) of biotinylated *atoD *probe were used in binding buffer consisting of 10 mM Tris-HCl pH 7.4, 50 mM NaCl, 1 mM EDTA, 4 mM DTT and 5% (v/v) glycerol, as previously described [5]. Competitive non-biotinylated *atoD *was added in the same concentration (up to 80 nM each) with *metR-metE, acrD *and *narG*. Each reaction took place in a final volume of 20 μl consisted of 0.2 mg/ml BSA and 2 μg sonicated calf thymus competitive DNA. All the ingredients were added and pre-incubated for 5 min at room temperature, before the addition of 0.35 μM (2 μg) of the protein and transfer to ice for 30 min. At the end of binding reactions, the samples were loaded to 4% (w/v) acrylamide gel in 0.25× TBE buffer and ran at 120 V, with a 30 min prerun at 100 V. DNA was transferred from the gels to Biodyne B membranes (Pall) and detected by Phototope-Star detection kit (New England Biolabs), as previously described [4,5].

## Abbreviations

AcAc: Acetoacetate; ChIP: Chromatin Immunoprecipitation; GO: Gene Ontology; GOT: Gene Ontology Term; LL: Log-Likelihood; ORF: Open Reading Frame; PSPM: Position-Specific Probability Matrix; TCS: Two-Component System; TFBS: Transcription Factor Binding Site; TSS: Transcription Start Site; EMSA: Electrophoretic Mobility Shift Assays.

## Authors' contributions

EP co-designed and carried out the computational analysis for *de novo *motif detection of putative AtoC binding targets within the *E. coli *genome workflow, participated in the interpretation of the results of the computational analysis, co-drafted and co-revised the manuscript, AC co-designed the computational analysis workflow for *de novo *motif detection of putative AtoC binding targets within the *E. coli *genome, supervised the computational analysis and interpretation of the results of the computational analysis, contributed in the evaluation of the results, co-drafted, co-revised and proofread the manuscript. AIG carried out the experimental assays (ChIP assays, Primer design and PCR validations, Electrophoretic Mobility Shift Assays) co-drafted and co-revised the manuscript, CAP supervised the experimental analysis and interpretation part, contributed in the evaluation of the results, co-drafted and co-revised the manuscript, FNK contributed in the supervision of the complete work, in the interpretation and evaluation of the results and revised the manuscript. DAK contributed in the supervision of the complete work, in the interpretation and functional evaluation of the results, co-drafted, co-revised and proofread the manuscript. All authors have read and approved the final manuscript.

## Supplementary Material

Additional file 1 **Motif matrices**. Text file with the position-specific probability matrices of motifs 1-5 in the INCLUSIVE [17] format. Click here for file

Additional file 2 **Results of the genome-wide screenings using the motifs 1-5**. Excel file with the motif searching results of each motif 1-5 at prior probability set to 0.7, in both intergenic and gene-encoding regions. Click here for file

Additional file 3 **Application of the computational procedure on the LexA transcription factor**. Excel file containing the results of the application of the computational procedure used in this work on LexA, a bacterial transcription factor that regulates many genes involved in SOS response [20]. Click here for file

Additional file 4 **Table including all primer sequences for binding site detection**. Text file with all the primers' sequences. Click here for file
