# Supplementary material for: Escherichia coli genome-wide promoter analysis: Identification of additional AtoC binding target elements
Source: BMC Genomics. 2011 May 13;12:238. doi: 10.1186/1471-2164-12-238 (PMC3118216; doi:10.1186/1471-2164-12-238)
Supplement: Additional file 4 — Table including all primer sequences for binding site detection. Text file with all the primers' sequences. [file 1471-2164-12-238-S4.DOC]

**Table 7 - Primer sequences for binding site detection**

**b)**

| **Primer names** | **Primer sequence information** | **Source or reference** |
| --- | --- | --- |
| UatoD | 5΄-ΤCGGΑΑΤΤCΑΤΤGΑΤGΤΑΤΑΑΑCΤCCΑGGΑΑ- 3΄ | [4] |
| LatoD | 5΄-ΑΑGGGΑΤCCGΤGGCGΤCΤTGTΑATGTCAΤ-3΄ | [4] |
| UdmsA | 5΄-AGTTCTGCGTCCGTATATGA-3΄ | This study |
| LdmsA | 5΄- CATAATGGCTCACTCAAGCT -3΄ | This study |
| UpuuP | 5΄-GGCCTGACATTTGCTGGTAAT-3΄ | This study |
| LpuuP | 5΄-ATTTTCGCAGACGGGTTTTG-3΄ | This study |
| UmetE | 5΄-TTCTCCCTGCGGTGTAAAGC-3΄ | This study |
| LmetE | 5΄-GTCATTTTTTCTTCCTCTAA-3΄ | This study |
| UbtuB | 5΄-GTCATCGCCATCGTGCCAGA-3΄ | This study |
| LbtuB | 5΄-GCAGTGTCCGCATAACGCAA-3΄ | This study |
| UykgQ | 5΄-TCATATCGCACATTTTCGTT-3΄ | This study |
| LykgQ | 5΄-TATTTTTACAGCAAGACAAC-3΄ | This study |
| UnarQ | 5΄-TCGGGGATGAAAATGGTGTT-3΄ | This study |
| LnarQ | 5΄-GCACAATGTAAAAAAAGGCC-3΄ | This study |
| UcpxP | 5΄-CAAGCAAAAGTAAATCAATG-3΄ | This study |
| LcpxP | 5΄-ATGACGGCAGCGGTAACTAT-3΄ | This study |
| UrtcR | 5΄-GCACGCCTTTGGTCCACATT-3΄ | This study |
| LrtcR | 5΄-ACTGTTTTACGCATCTTAG -3΄ | This study |
| UymiA | 5΄- TTTCTTGCGTTATTTTCGGC -3΄ | This study |
| LymiA | 5΄- GCGGCGGGGTTCCTGATTTC -3΄ | This study |
| UnarZ | 5΄- GTTTATGGTCGGCGGAAGTT -3΄ | This study |
| LnarZ | 5΄- GGTAGTCGGTCTGTTGGATT -3΄ | This study |
| Ucrr | 5΄- TTGCTGGGGATGGGTCTGGA -3΄ | This study |
| Lcrr | 5΄- CGGTATCCTTCTTGTCGTCG -3΄ | This study |
| UfliT | 5΄-CAGCGAAATGGCGTATGTGA-3΄ | This study |
| LfliT | 5΄-CATTCAAGGGGAACATTAGA-3΄ | This study |
| UnarG | 5΄-ACTTTATTTTTCATCCCCAT-3΄ | This study |
| LnarG | 5΄-GGTAGAGCGGACGATTTTGT-3΄ | This study |
| UycjM | 5΄-AGTTTATTTTCTGCGGAGTA-3΄ | This study |
| LycjM | 5΄-TAACACGACATCACTTTCAT-3΄ | This study |
| UyeaM | 5΄-AAGAGACGGGGTTGAGTTTT-3΄ | This study |
| LyeaM | 5΄-GGTTTCGCTGATTGTGGGGA-3΄ | This study |
| UgadA | 5΄-GCTGGGTTATCTGGCGTGAC-3΄ | This study |
| LgadA | 5΄-CTTCACCATCTTTCAGTTTG-3΄ | This study |
| UeutR | 5΄-GTGCGAAAAGTGCTGGGGGA-3΄ | This study |
| LeutR | 5΄-GGCGTTCAGGCGAATGCGT-3΄ | This study |
| UnirB | 5΄- CGCTGTCGTCTTTGTGATGT -3΄ | This study |
| LnirB | 5΄- GATTTCTTTTCTATTACCGC -3΄ | This study |
| UykgE | 5΄- GCACCCTGAATAAAACCGCC -3΄ | This study |
| LykgE | 5΄- TACAAAAAAATCACCGACAA -3΄ | This study |
| UborD | 5΄- GCAGAGAAGTTCCCCGTCAG -3΄ | This study |
| LborD | 5΄- GCAGTAGCGAGTAGCATTTT -3΄ | This study |
| UputP | 5΄-GCAACCGCAAAAAATGTGAGAG-3΄ | This study |
| LputP | 5΄-ATATCCGACGCACCCGCCGA-3΄ | This study |
| UacrD | 5΄-GCGGAACGGCTAGGTGGGAC-3΄ | This study |
| LacrD | 5΄- CATTAAAAGAGGACCTCGTG -3΄ | This study |
| UycjH | 5΄-CGACTAACTCCCTGAAATGC-3΄ | This study |
| LycjH | 5΄-GCTGATTGAGGGACTTTACG-3΄ | This study |
